# The Preparation of Hot Compressed Air

**Published:** 1908-08-01

**Authors:** 


### Therapeutics.

### THE PREPARATION OF HOT COMPRESSED AIR.

In The Hospital of May 23rd, 1908, a description is given of the method employed by M. Prat, of preparing hot compressed air, for surgical purposes. M. Prat takes the air already compressed and heats it by passing the air over platinum wires, kept incandescent by a current of carburetted vapour. It appears to the writer that the method employed for preparing the air is a most unnecessarily complicated one. When air is compressed, it is heated, as a matter of course, in the act of compression, and therefore any temperature can be obtained by the process of compression. The process of compressing air is a very simple one. Power is expended in compressing the air, just as in driving the plunger of a pump, and the whole of the power, except that taken to overcome the friction of the plunger and working parts of the pump, is converted into heat in the air itself. As the pressure increases, the temperature of the air rises. Thus with air at an initial temperature of 60° F. at ordinary atmospheric pressure, when the pressure is two atmospheres, the temperature is about 160° F. (71.1° C.), at three atmospheres it is about 250° F. (121.1° C.), at four atmospheres it is about 320° F. (160° C.), at six atmospheres 430° F. (221.1° C.), and at ten atmospheres 560° F. (293.3° C.), the temperature rising with increasing rapidity, as the pressure increases. In the ordinary working of compressed air apparatus the heat that is liberated in the air is got rid of, but in the present instance the method to be adopted would be to store the heat as it is produced, so that the compressed air would be at the temperature required. The heated compressed air could be produced as and when required by the employment of a small compressor, constructed for the purpose, and driven by an electric motor taking current from the hospital electricity service. The temperature and pressure of the air when in use could be regulated by means of the valve employed for controlling the egress of the air.

Where it is not convenient to compress the air at the hospital, the air could be compressed by an insulated compressor, and delivered into one of the steel bottles employed for the carriage of compressed air and gases, the bottle and its accessories being also insulated.

